# Targetable driver gene–tumor immune microenvironment axis in non-small cell lung cancer: from molecular pathological mechanisms to precision immunotherapy stratification strategies

**DOI:** 10.3389/fimmu.2026.1801943

**Published:** 2026-04-16

**Authors:** Wenqing Chen, Fengyan Zhang, Xin Zheng

**Affiliations:** 1 Department of Internal Medicine, Affiliated Hospital of Shandong University of Traditional Chinese Medicine, Jinan, Shandong, China; 2 Department of Oncology, The Second Affiliated Hospital of Shandong University of Traditional Chinese Medicine, Jinan, Shandong, China; 3 Department of Internal Medicine, Qingdao Traditional Chinese Medicine Hospital (Haici Hospital), Qingdao, Shandong, China

**Keywords:** immune checkpoint inhibitors, non–small cell lung cancer, precision immunotherapy, targetable driver genes, tumor immune microenvironment

## Abstract

Non-small cell lung cancer (NSCLC) is characterized by substantial molecular heterogeneity that critically influences the efficacy of immunotherapy. Although immune checkpoint inhibitors (ICIs) have improved outcomes in selected patients, responses vary markedly across molecular subtypes defined by targetable driver gene alterations. Increasing evidence indicates that oncogenic drivers, including *EGFR*, *ALK*, *KRAS*, *MET*, *RET*, and *BRAF*, actively shape the tumor immune microenvironment (TIME) by regulating antigen presentation, immune cell infiltration, cytokine signaling, metabolic programs, and immune checkpoint expression. These interactions generate distinct driver gene–associated immune phenotypes that underlie differential sensitivity and resistance to ICIs. Recent advances in single-cell and spatial profiling have further revealed the complexity and spatial organization of these immune landscapes. In this review, we summarize current mechanistic and clinical evidence supporting the targetable driver gene–TIME axis in NSCLC and discuss its implications for immunotherapy response, resistance, and patient stratification. This integrative framework provides a rationale for precision immunotherapy strategies and the design of biomarker-driven clinical trials.

## 1 Introduction

NSCLC accounts for approximately 85% of all lung cancer cases and remains the leading cause of cancer-related mortality worldwide (1). Despite substantial progress in early diagnosis, surgical techniques, and systemic therapies, the long-term survival of patients with advanced or metastatic NSCLC remains unsatisfactory (2). This unfavorable prognosis largely reflects the profound molecular, cellular, and immunological heterogeneity that characterizes NSCLC, resulting in highly variable therapeutic responses and frequent treatment resistance (3). Over the past two decades, the molecular stratification of NSCLC based on oncogenic driver alterations has fundamentally transformed disease classification and management, shifting clinical practice from histology-based treatment toward precision oncology.

The discovery of actionable driver genes—including EGFR, ALK, ROS1, RET, MET, BRAF, and KRAS—has enabled the development of targeted therapies that achieve high initial response rates and meaningful survival benefits in selected patient populations (4). Nevertheless, the durability of these responses is limited by the inevitable emergence of acquired resistance, and subsequent therapeutic options remain suboptimal for many patients. In parallel, ICIs targeting the PD-1/PD-L1 axis have revolutionized the treatment paradigm of NSCLC, offering durable responses and long-term survival in a subset of patients, particularly those without targetable driver alterations. However, the clinical efficacy of ICIs is strikingly heterogeneous, with response rates varying widely across molecular subtypes and clinical contexts (5, 6).

Importantly, accumulating clinical evidence suggests that patients harboring oncogenic driver alterations—most notably EGFR mutations and ALK rearrangements—tend to derive relatively limited benefit from ICIs, even in the presence of PD-L1 expression (7). This apparent paradox underscores a fundamental limitation of relying on single biomarkers such as PD-L1 or tumor mutational burden (TMB) to predict immunotherapy response. Indeed, although PD-L1 expression and TMB have been incorporated into clinical decision-making, they only partially reflect the complexity of the tumor immune microenvironment (TIME), which encompasses not only immune cell density but also spatial organization, functional states, metabolic constraints, and dynamic interactions between tumor and immune compartments (8, 9).

Recent advances in cancer immunology have shifted attention toward the TIME as a central determinant of immunotherapy efficacy. The TIME is increasingly recognized as a highly dynamic ecosystem shaped by tumor-intrinsic genetic alterations and extrinsic microenvironmental cues (10, 11). In NSCLC, distinct immune phenotypes—ranging from immune-desert and immune-excluded tumors to inflamed but functionally suppressed microenvironments—have been described, each associated with differential sensitivity to immunotherapeutic interventions. Notably, emerging evidence suggests that these immune states are not randomly distributed but are, at least in part, orchestrated by oncogenic driver signaling (12, 13).

Mechanistic and translational studies now demonstrate that targetable driver genes extend their influence beyond tumor cell proliferation and survival to actively remodel the immune landscape (14). Oncogenic pathways driven by EGFR, ALK, and KRAS have been reported to regulate antigen presentation machinery, modulate cytokine and chemokine secretion, alter immune checkpoint expression, reprogram tumor and immune cell metabolism, and influence the recruitment and functional polarization of immune effector and suppressor populations (14–16). Through these coordinated mechanisms, driver gene alterations give rise to distinct driver-associated TIME configurations that critically shape both primary resistance and acquired resistance to ICIs, as well as the outcomes of combination strategies involving targeted agents and immunotherapy (17–19).

The rapid evolution of high-resolution profiling technologies has further deepened our understanding of the interplay between oncogenic signaling and tumor immunity. Single-cell RNA sequencing, spatial transcriptomics, multiplex immunofluorescence, and digital pathology have revealed previously unrecognized heterogeneity in immune cell composition, spatial architecture, and cell–cell communication within NSCLC tumors (20, 21). These approaches have uncovered complex, driver gene–specific immune niches and ligand–receptor interaction networks that cannot be captured by bulk analyses alone, reinforcing the need for integrative frameworks that bridge molecular pathology and immune contexture.

Against this background, the concept of a “targetable driver gene–tumor immune microenvironment axis” has emerged as a unifying paradigm to explain the divergent immunotherapy outcomes observed across NSCLC molecular subtypes. Rather than viewing oncogenic drivers and immune features as independent determinants, this axis emphasizes their bidirectional and dynamic interdependence (22, 23). Elucidating this axis has profound clinical implications, as it may inform patient selection, guide therapeutic sequencing, identify rational combination strategies, and support the development of biomarker-driven clinical trials tailored to specific molecular–immune contexts.

In this review, we comprehensively synthesize current molecular, immunological, and clinical evidence to delineate the pathological basis and mechanistic underpinnings of the targetable driver gene–TIME axis in NSCLC. We critically examine how distinct driver alterations shape immune phenotypes, influence immunotherapy response and resistance, and interact with emerging treatment modalities. Finally, we discuss how integrating driver gene status with TIME features may enable more precise immunotherapy stratification and foster the development of personalized treatment strategies for patients with NSCLC.

## 2 Targetable driver genes and their molecular characteristics in non–small cell lung cancer

**Figure 1:**
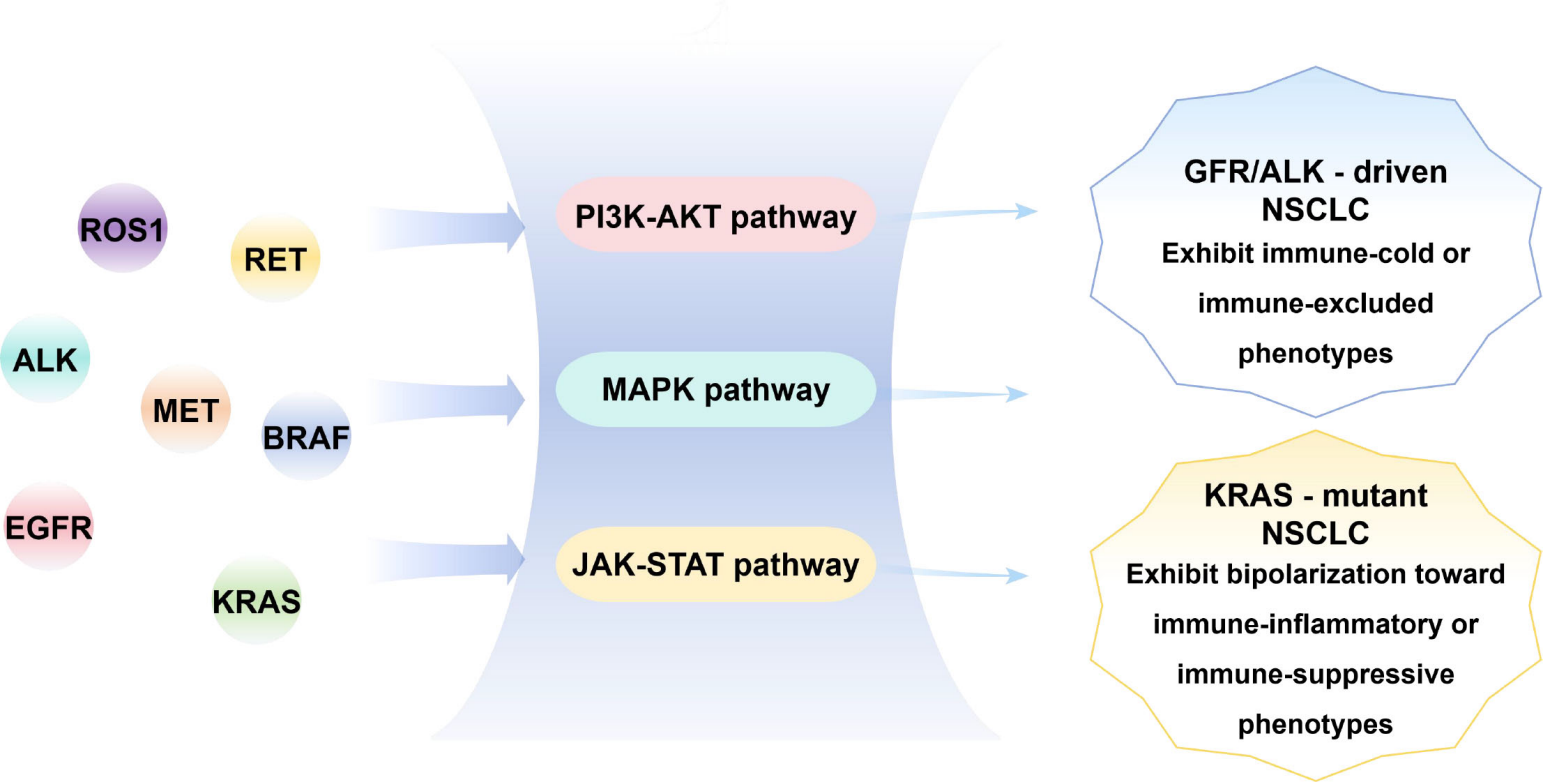
Spectrum of targetable driver genes and their major molecular pathological features in non–small cell lung cancer.

NSCLC is now widely recognized as a molecularly heterogeneous disease in which distinct oncogenic driver alterations, rather than histology alone, define tumor biology and therapeutic vulnerability. Large-scale genomic profiling efforts have identified a set of recurrent, clinically actionable driver genes, including EGFR, ALK, ROS1, RET, BRAF, MET, and KRAS. These alterations differ markedly in mutation or fusion patterns, demographic distribution, histological associations, and downstream signaling consequences, collectively forming the molecular foundation of precision oncology in NSCLC. An overview of the major targetable driver genes, their common alteration types, principal signaling pathways, and global immune-related trends is summarized in **Figure 1**.

### 2.1 Major targetable driver genes and their clinicopathological characteristics

Activating EGFR mutations represent one of the most prevalent oncogenic drivers in NSCLC, particularly in lung adenocarcinoma. Canonical exon 19 deletions and exon 21 L858R substitutions account for the majority of cases and are strongly enriched in never-smokers, females, and East Asian populations (24). These alterations confer marked sensitivity to EGFR tyrosine kinase inhibitors (TKIs), ranging from first-generation reversible inhibitors to third-generation agents designed to overcome resistance mutations such as T790M. Clinical trial data have shown that second-generation EGFR TKIs can improve progression-free survival compared with first-generation TKIs, with treatment benefit varying across mutation subtypes (often more pronounced in Ex19del than in L858R) (25, 26). Despite high initial response rates, most tumors eventually develop acquired resistance, underscoring the need for complementary therapeutic strategies (27).

Rearrangements involving ALK occur in approximately 3–7% of NSCLC cases and are most commonly represented by EML4–ALK fusions. These tumors typically arise in younger patients with adenocarcinoma histology and minimal smoking exposure. Similar chromosomal rearrangements define ROS1- and RET-positive NSCLC, which together account for a small but clinically meaningful subset of patients (28, 29). RET fusion has been found in 1% to 2% of NSCLC patients, which is associated with a high risk of brain metastasis (30). The development of highly selective next-generation inhibitors targeting these fusion proteins has substantially improved clinical outcomes, highlighting the strong oncogenic dependency of these tumors. Importantly, oncogene-addicted NSCLC often exhibits distinct immune phenotypes that may influence responsiveness to immune checkpoint blockade, motivating integrated gene-TIME stratification in subsequent sections.

BRAF mutations are detected in a minority of NSCLC cases, with BRAF V600E being the most clinically actionable variant. Unlike EGFR and ALK alterations, BRAF mutations are observed in both smokers and never-smokers and exhibit greater biological heterogeneity. Alterations in MET, particularly exon 14 skipping mutations and gene amplification, define another distinct targetable subgroup, often enriched in older patients and associated with aggressive clinical behavior (31, 32).

In contrast, KRAS mutations represent the most common oncogenic driver events in NSCLC, especially among smokers. For decades, KRAS was considered undruggable; however, the development of covalent inhibitors targeting KRAS G12C has substantially changed therapeutic options for this molecular subset (33). Importantly, KRAS-mutant tumors display substantial molecular diversity driven by co-occurring genetic alterations, which can shape downstream signaling programs and tumor immune phenotypes—an important consideration for gene–TIME–integrated stratification (**Figure 1**).

### 2.2 Key signaling pathways and tumor biological behavior

At the molecular level, targetable driver genes exert their oncogenic effects primarily through constitutive activation of conserved intracellular signaling cascades that regulate tumor cell proliferation, survival, and dissemination. Receptor tyrosine kinase (RTK) drivers such as EGFR, ALK, ROS1, and RET converge on key downstream pathways, including PI3K–AKT, MAPK, and JAK–STAT, enabling sustained growth signaling and resistance to apoptosis, as illustrated in the central panel of **Figure 1**.

The PI3K–AKT pathway plays a pivotal role in controlling cellular metabolism, survival, and protein synthesis. Preclinical mechanistic studies have shown that persistent activation of this pathway not only supports tumor growth but also influences immune-related processes, including antigen processing and major histocompatibility complex (MHC) class I expression (34, 35). Dysregulation of antigen presentation machinery can reduce tumor immunogenicity and impair effective immune recognition.

The MAPK cascade, particularly the RAS–RAF–MEK–ERK axis, is a dominant driver of oncogenic signaling in KRAS-mutant NSCLC. Continuous MAPK activation promotes cell cycle progression, invasion, and metastatic potential, while simultaneously modulating transcriptional programs that regulate tumor growth and immune-related pathways, as demonstrated in mechanistic studies of MAPK signaling in cancer (36). Evidence from experimental tumor models and transcriptomic analyses suggests that hyperactive MAPK signaling may suppress interferon responses and downregulate genes involved in antigen presentation, thereby contributing to immune evasion (37–39).

The JAK–STAT pathway, frequently engaged downstream of RTKs, integrates oncogenic and inflammatory cues and regulates cytokine production, immune checkpoint expression, and tumor–immune crosstalk. Mechanistic studies of JAK–STAT signaling in immune regulation have shown that aberrant activation of this pathway can foster an immunosuppressive microenvironment by shaping cytokine networks and promoting recruitment of regulatory immune populations. Collectively, these signaling pathways extend beyond tumor-intrinsic biology to actively interact with immune regulatory mechanisms, reinforcing the concept that oncogenic signaling and immune escape are mechanistically intertwined rather than independent processes (40).

### 2.3 Targetable driver genes and global trends in classical immune biomarkers

Clinical and translational studies have revealed distinct yet partially overlapping patterns of classical immune biomarkers across different driver gene subtypes, as summarized in the right panel of **Figure 1**. In general, EGFR- and ALK-driven NSCLC is characterized by relatively low TMB, limited neoantigen diversity, and sparse infiltration of cytotoxic T lymphocytes (41). Consistently, the median density of CD8^+^ tumor-infiltrating lymphocytes was significantly lower in EGFR-mutant than in KRAS-mutant tumors (185.1 vs. 330.1 cells/mm^2^, P = 0.011) (42). Although PD-L1 expression may still be detectable, this does not necessarily translate into effective immune checkpoint blockade. In treatment-naïve EGFR-mutant tumors, PD-L1 positivity was observed in 24%, 16%, and 11% of cases at cutoffs of ≥1%, ≥5%, and ≥50%, respectively, while the corresponding rates in ALK-rearranged tumors were 63%, 47%, and 26%. Nevertheless, the objective response rate to PD-1/PD-L1 inhibitors in EGFR-mutant/ALK-positive NSCLC was only 3.6% (1/28), compared with 23.3% (7/30) in EGFR wild-type/ALK-negative tumors (42).

By contrast, KRAS-mutant NSCLC—particularly tumors harboring concurrent TP53 mutations—often exhibits a more inflamed immune phenotype characterized by increased cytotoxic T-cell infiltration and enhanced responsiveness to immune checkpoint inhibitors. In patients with lung adenocarcinoma treated with PD-1 blockade, KRAS–TP53 co-mutated tumors demonstrated an objective response rate of 57.1% in an early cohort analysis, compared with 28.6% in KRAS-only tumors and 7.7% in KRAS/TP53 wild-type tumors, with a median PFS of approximately 8.5 months (43). However, this favorable immune context is not universal. KRAS tumors harboring STK11 or KEAP1 co-mutations frequently display immune-excluded phenotypes and primary resistance to immunotherapy, with objective response rates of approximately 7.4% following PD-1 blockade in KRAS-mutant lung adenocarcinoma cohorts (44).

Collectively, these observations suggest that targetable driver genes are associated with distinct baseline immune landscapes characterized by differences in PD-L1 expression, mutational load, and immune cell composition (45). As conceptualized in **Figure 1**, these molecularly driven immune trends provide a critical pathological foundation for subsequent classification of immune-inflamed, immune-cold, and immune-excluded tumors, setting the stage for a more nuanced understanding of the tumor immune microenvironment and its therapeutic implications in NSCLC.

## 3 The targetable driver gene–tumor immune microenvironment axis: key mechanisms and immune landscapes

**Figure 2:**
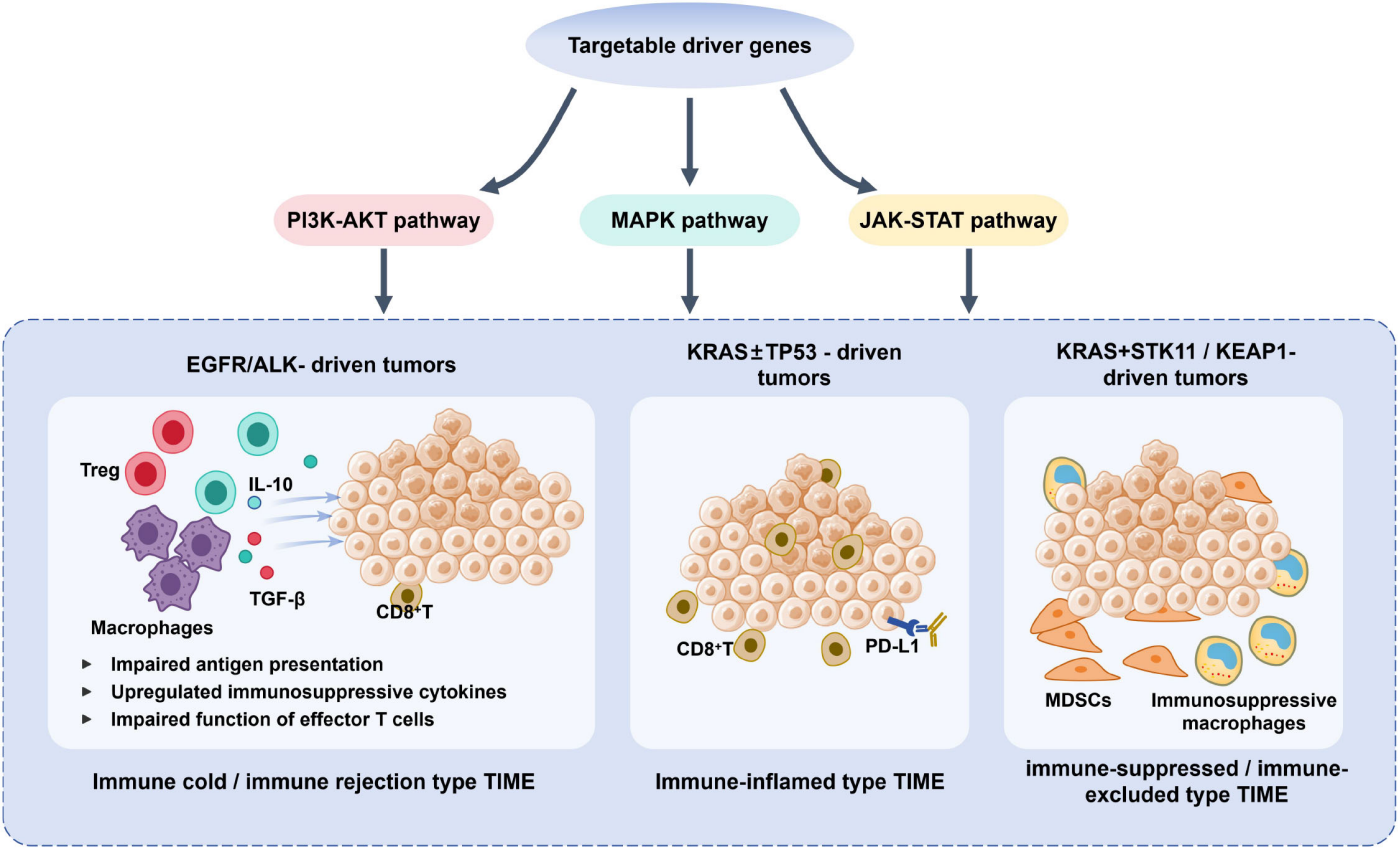
Mechanistic schematic of the targetable driver gene-TIME axis in non-small cell lung cancer.

Accumulating mechanistic and translational evidence suggests that targetable oncogenic driver alterations in NSCLC influence not only tumor-intrinsic growth signaling but also the composition, spatial organization, and functional state of the TIME (46). These effects appear to be mediated through coordinated modulation of oncogenic signaling pathways, antigen presentation, cytokine networks, and immune cell recruitment (42). An integrated overview of these interactions is schematically illustrated in **Figure 2**, which depicts how distinct driver gene contexts program divergent immune phenotypes along the driver gene–TIME axis.

### 3.1 EGFR/ALK-driven tumors and the immune-cold or immune-excluded TIME

Tumors driven by kinase-type oncogenic alterations, most notably EGFR mutations and ALK rearrangements, are generally associated with relatively unfavorable TIME features and limited clinical benefit from ICIs (47). As summarized in **Figure 2**, these tumors preferentially activate downstream PI3K–AKT, MAPK, and JAK–STAT signaling cascades, which collectively contribute to immune evasion through multiple convergent mechanisms.

At the genomic level, EGFR- and ALK-driven NSCLC typically exhibits a low TMB, resulting in reduced neoantigen generation and limited baseline immunogenicity. Beyond mutational load, previous mechanistic and translational studies summarized in recent reviews suggest that oncogenic EGFR and ALK signaling may impair antigen presentation by downregulating major histocompatibility complex class I (MHC-I) molecules and components of the antigen-processing machinery (48, 49). This reduction in antigen visibility constrains effective priming and recognition of tumor-specific T cells.

Concurrently, EGFR/ALK-driven signaling actively shapes an immunosuppressive cytokine milieu. Experimental studies in NSCLC models have shown that oncogenic EGFR signaling can induce the upregulation of immunosuppressive mediators such as transforming growth factor–β (TGF-β), which suppresses cytotoxic CD8^+^ T-cell activity and contributes to resistance to immune checkpoint blockade (50). More broadly, immunosuppressive cytokines such as TGF-β and interleukin-10 (IL-10) have been shown in experimental and translational studies to promote the expansion of regulatory T cells and M2-polarized tumor-associated macrophages, thereby reinforcing an immunosuppressive tumor microenvironment (51, 52). As illustrated in **Figure 2**, these combined effects result in either an immune-cold phenotype, characterized by sparse immune infiltration, or an immune-excluded phenotype, in which immune cells accumulate at the tumor margin but fail to penetrate the tumor parenchyma.

Importantly, the TIME in EGFR/ALK-driven tumors is dynamic rather than static. TKI therapy can transiently enhance tumor antigen release and inflammatory signaling, occasionally increasing immune cell infiltration during early treatment phases (53). However, this immunostimulatory window is often short-lived and counterbalanced by adaptive upregulation of immunosuppressive pathways, including PD-L1 expression and TGF-β signaling, particularly at the emergence of acquired resistance. These temporal dynamics, summarized in **Figure 2**, underscore the complexity of integrating immunotherapy into kinase-driven disease and highlight the need for precise biological and temporal stratification.

### 3.2 KRAS and co-mutation–dependent heterogeneity of the TIME

In contrast to kinase-driven tumors, KRAS-mutant NSCLC displays pronounced heterogeneity in immune landscapes, largely dictated by co-occurring genetic alterations. As depicted in **Figure 2**, KRAS functions as a central signaling hub linking oncogenic activation of the RAS–RAF–MEK–ERK pathway to divergent immune outcomes.

Tumors harboring KRAS mutations alone or in combination with TP53 frequently exhibit immune-inflamed (“immune-hot”) TIME features. These tumors are characterized by higher TMB, robust infiltration of CD8^+^ cytotoxic T lymphocytes, activation of interferon-related and inflammatory pathways, and increased expression of immune checkpoints (54). Functionally, KRAS–TP53 co-mutated tumors often demonstrate preserved antigen presentation and heightened immune surveillance, reflecting a relatively inflamed tumor microenvironment (55). Consistent with these immune-active features, clinical cohorts have reported higher response rates to ICIs in KRAS–TP53 co-mutated tumors, with objective response rates of approximately 35–36% following PD-1 blockade in KRAS-mutant NSCLC cohorts in larger retrospective clinical cohorts, depending on cohort and treatment setting (44).

By contrast, KRAS tumors co-mutated with STK11 (LKB1) or KEAP1 represent prototypical immunotherapy-resistant subtypes. Previous mechanistic studies, as summarized in recent reviews, indicate that loss of STK11 disrupts cellular energy sensing and suppresses AMP-activated protein kinase (AMPK) signaling, leading to metabolic reprogramming that is unfavorable for effective T-cell activation and persistence (56). These tumors exhibit reduced CD8^+^ T-cell infiltration, enrichment of myeloid-derived suppressor cells (MDSCs), and attenuated interferon signaling, resulting in immune-excluded or immune-suppressed TIME states.

Similarly, mechanistic and translational studies summarized in recent reviews indicate that KEAP1 co-mutation can lead to persistent activation of the NRF2 antioxidant pathway, conferring resistance to oxidative stress and reinforcing immune evasion through metabolic and redox remodeling (57, 58). As highlighted in **Figure 2**, these co-mutation patterns emphasize that KRAS mutation alone does not determine immune behavior; rather, the combinatorial genetic context dictates whether the TIME is inflamed and immunotherapy-sensitive or suppressive and resistant.

### 3.3 Single-cell and spatial omics reveal the fine architecture of driver-associated TIME

Recent advances in single-cell RNA sequencing (scRNA-seq), spatial transcriptomics, and multiplexed imaging technologies have provided high-resolution insights into the cellular and spatial organization of driver-associated TIME heterogeneity (59). These approaches demonstrate that immune composition and functional states vary not only across molecular subtypes but also across distinct intratumoral regions, thereby refining the mechanistic framework illustrated in **Figure 2**.

Single-cell analyses reveal that EGFR- and ALK-driven tumors are enriched for immunosuppressive myeloid populations and dysfunctional or exhausted T-cell states, whereas KRAS–TP53 tumors harbor expanded populations of effector CD8^+^ T cells and pro-inflammatory macrophages (60). Spatial transcriptomic profiling further identifies marked compartmentalization of immune cells, with immune exclusion frequently observed at the invasive front of kinase-driven tumors.

At the level of intercellular communication, ligand–receptor interaction analyses have uncovered driver-specific signaling networks that orchestrate immune cell trafficking and suppression. Chemokine axes such as CXCL–CXCR regulate immune cell recruitment, while TGF-β signaling emerges as a dominant suppressive pathway mediating immune exclusion and fibroblast–immune crosstalk (61). These spatially resolved interaction patterns provide mechanistic support for the driver gene–TIME relationships summarized in **Figure 2** and enable the construction of integrated “gene–TIME maps” that extend beyond bulk biomarker analyses.

Collectively, the available evidence supports a unifying model in which targetable driver genes reshape the tumor immune microenvironment through coordinated modulation of oncogenic signaling pathways, cytokine networks, metabolic programs, and immune cell recruitment. As summarized in **Figure 2**, kinase-driven alterations such as EGFR and ALK preferentially give rise to immune-cold or immune-excluded TIME states, whereas KRAS mutations generate a spectrum of immune phenotypes that are highly dependent on co-mutation context.

Based on these patterns, several representative “driver gene–TIME subtypes” can be delineated, including immune-cold kinase-driven tumors, immune-inflamed KRAS–TP53 tumors, and immunosuppressive KRAS–STK11/KEAP1 tumors. Although the driver gene–TIME axis described above primarily provides a conceptual framework, several widely used clinical biomarkers can offer practical operational guidance for classifying these immune phenotypes. In clinical and translational studies, high PD-L1 expression is commonly defined as a tumor proportion score (TPS) ≥50%, while tumors with TPS <1% are often considered PD-L1–negative (62, 63). Similarly, TMB ≥10 mutations/Mb is frequently used as a threshold for TMB-high status, which has been associated with increased neoantigen load and enhanced responsiveness to immune checkpoint inhibitors (64, 65). In addition, immune-inflamed tumors are typically characterized by abundant intratumoral CD8^+^ T-cell infiltration, whereas immune-excluded tumors display immune cell accumulation at the tumor margin without effective penetration into tumor parenchyma (10). This mechanistic framework provides a biological foundation for subsequent immunotherapy stratification and underscores the necessity of integrating molecular drivers with immune contexture in precision treatment strategies.

## 4 The targetable driver gene–TIME axis in immunotherapy response, resistance, and preliminary stratification

**Figure 3:**
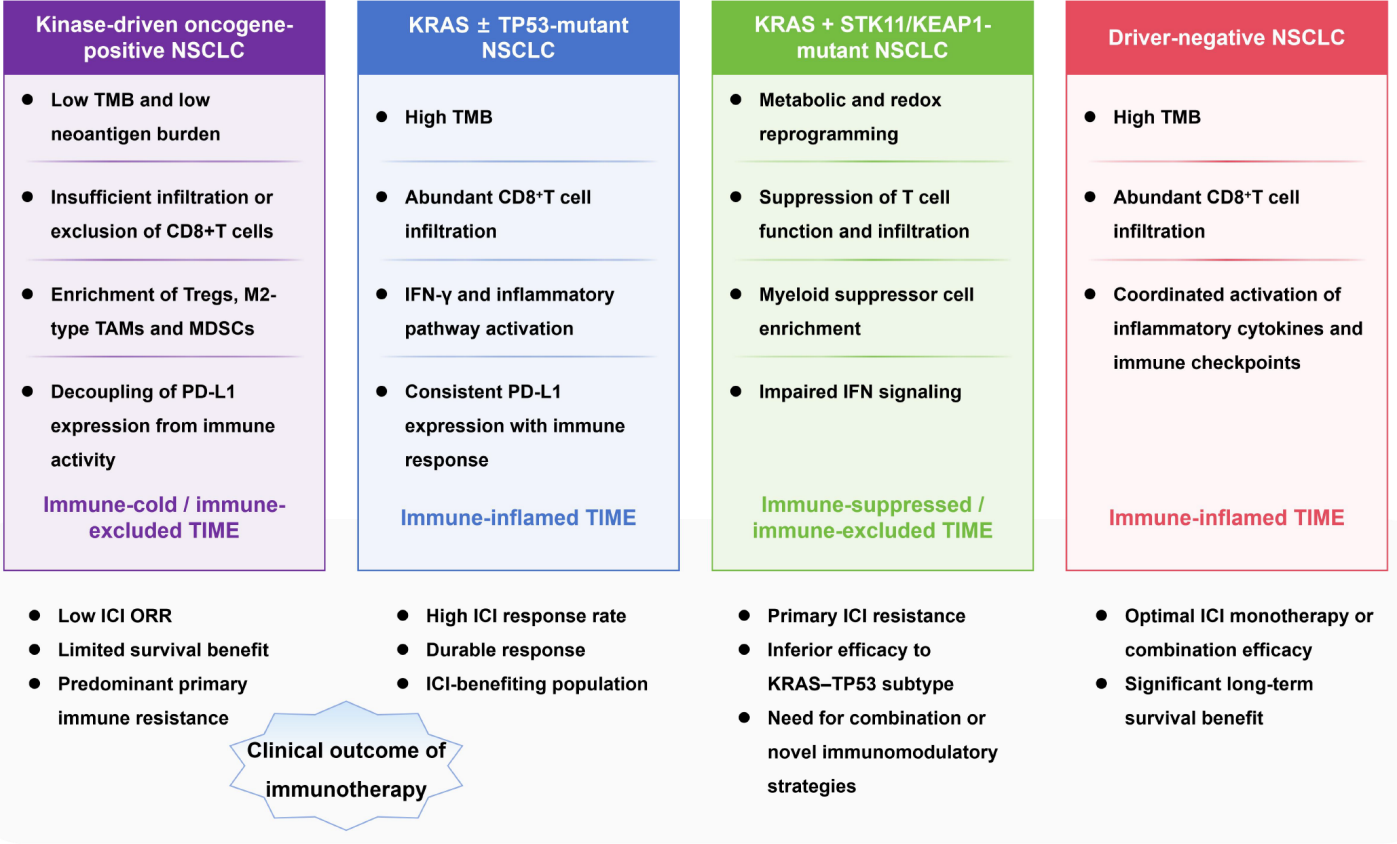
Relationship between targetable driver gene-defined subtypes, tumor immune microenvironment phenotypes, and immunotherapy outcomes in non-small cell lung cancer.

ICI efficacy in NSCLC displays marked heterogeneity that cannot be adequately explained by any single molecular or immune biomarker. Increasing evidence indicates that immunotherapy outcomes are determined by the coordinated interaction between oncogenic driver alterations and the TIME (66). To conceptualize this interaction, **Figure 3** summarizes the relationship between major targetable driver gene subtypes, dominant TIME phenotypes, and characteristic patterns of immunotherapy response and resistance, providing an integrated framework for the clinical observations discussed below.

### 4.1 Differential immunotherapy outcomes across targetable driver gene subtypes

Clinical trials and real-world studies suggest that ICI efficacy varies substantially according to targetable driver gene status. In most studies, tumors harboring kinase-type oncogenic drivers—particularly EGFR mutations—demonstrate low response rates and limited survival benefit from ICI monotherapy. For example, in EGFR-mutated NSCLC, reported objective response rates (ORRs) were generally 4–14%, with median PFS of approximately 1.8–3.0 months in retrospective or early-phase studies. Pooled analyses of randomized trials further showed no significant overall survival benefit compared with docetaxel (HR 1.05–1.12, 95% CI 0.69–1.59 to 0.80–1.56) (7, 67). These unfavorable outcomes are observed across treatment lines and persist even in tumors with measurable PD-L1 expression, underscoring the intrinsic resistance of these molecular subtypes to immune checkpoint blockade.

As illustrated in **Figure 3**, EGFR or ALK-driven tumors predominantly map to immune-cold or immune-excluded TIME states, which are characterized by poor immune cell infiltration and limited antitumor immune activity. In contrast, driver-negative NSCLC, particularly smoking-associated tumors, often exhibits immune-inflamed microenvironments and derives more consistent benefit from ICIs, either as monotherapy or in combination with chemotherapy and/or antiangiogenic agents.

Within the spectrum of driver-positive disease, KRAS-mutant NSCLC represents a biologically and clinically heterogeneous group (68). Accumulating clinical evidence indicates that KRAS mutations, especially KRAS G12C, do not inherently preclude immunotherapy benefit. Indeed, KRAS single-mutant tumors—particularly those co-mutated with TP53—often demonstrate objective responses and durable disease control with ICIs. For example, a clinical study reported an ORR of approximately 26.7% and a disease control rate (DCR) of 73.3% in KRAS-mutant NSCLC treated with immunotherapy, with improved outcomes observed in tumors harboring KRAS–TP53 co-mutations (69). Conversely, certain co-mutation contexts (e.g., STK11/KEAP1) have been associated with immune-cold phenotypes and reduced ICI responsiveness, underscoring the need for co-mutation-aware stratification. Reflecting these patterns, current clinical guidelines generally discourage ICIs monotherapy in EGFR- or ALK-driven NSCLC, while endorsing ICIs as a standard component of treatment for driver-negative and selected KRAS-mutant populations.

### 4.2 Gene–TIME determinants of immunotherapy resistance

Beyond observed differences in clinical efficacy, mechanistic studies increasingly suggest that immunotherapy resistance in NSCLC is shaped by distinct gene–TIME configurations, rather than by oncogenic drivers in isolation. In EGFR- and ALK-driven tumors, primary resistance to ICIs is associated with a convergence of immune-evasive features, including low infiltration of cytotoxic CD8^+^ T cells, impaired antigen presentation due to downregulation of major histocompatibility complex (MHC) class I molecules, and enrichment of immunosuppressive cell populations such as regulatory T cells, MDSCs, and M2-polarized tumor-associated macrophages (50). These features may collectively contribute to T-cell dysfunction and impaired interferon signaling, thereby limiting effective antitumor immunity.

A parallel but mechanistically distinct resistance pattern has been reported in KRAS-mutant tumors harboring co-mutations in STK11 or KEAP1. As positioned in **Figure 3**, these tumors occupy immunosuppressive or immune-excluded TIME compartments. Loss of STK11 disrupts cellular energy sensing and promotes metabolic reprogramming that constrains T-cell activation and persistence, while KEAP1 mutations drive NRF2-mediated antioxidant responses that further reinforce immune escape (70). The resulting TIME is characterized by reduced effector T-cell activity, expansion of suppressive myeloid populations, altered nutrient availability, and resistance to immune-mediated cytotoxicity.

Across these molecular contexts, a consistent pattern emerges in which oncogenic signaling, immune suppression, and metabolic dysregulation converge to establish a permissive environment for immune evasion. This integrated “gene–TIME–immune resistance” axis provides a conceptual framework for understanding the limited efficacy of ICIs in specific NSCLC subtypes.

### 4.3 A preliminary stratification model based on the targetable driver gene–TIME axis

Building on these observations, a preliminary yet clinically meaningful stratification model can be proposed by jointly considering targetable driver gene status and dominant TIME phenotypes, as summarized in **Figure 3**. This two-dimensional framework emphasizes that immunotherapy outcomes are not dictated by genetic or immune features in isolation, but by their integrated positioning along the driver gene–TIME axis.

At a conceptual level, kinase-driven tumors (EGFR, ALK, ROS1, RET) typically correspond to immune-cold or immune-excluded TIME states and demonstrate limited sensitivity to ICIs, supporting a treatment strategy centered on targeted therapy (71). In contrast, KRAS single-mutant tumors, particularly those co-mutated with TP53, frequently display immune-inflamed microenvironments and represent suitable candidates for ICI-based regimens (72). By comparison, KRAS tumors harboring STK11 or KEAP1 co-mutations exemplify an immunosuppressive and metabolically constrained TIME, in which standard immunotherapy is often ineffective and combination strategies may be required.

Finally, driver-negative tumors with high tumor mutational burden and inflamed immune landscapes constitute the prototypical immunotherapy-responsive population, for whom ICIs form the therapeutic backbone. Collectively, this stratification underscores the clinical relevance of the targetable driver gene–TIME axis and provides a conceptual bridge toward more refined and actionable immunotherapy decision-making frameworks (73).

## 5 Clinical application and stratification of immunotherapy in NSCLC based on the targetable driver gene–TIME axis

**Figure 4:**
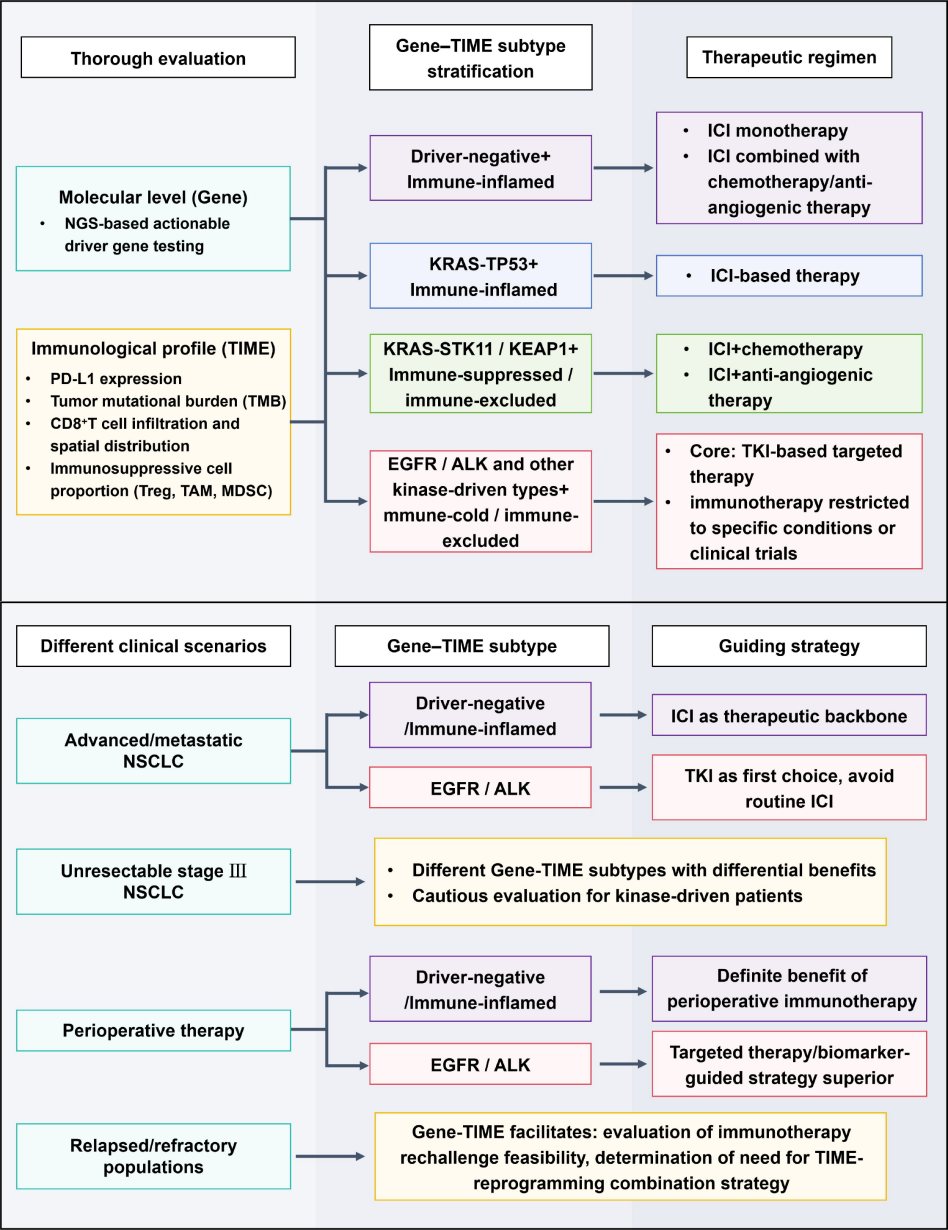
Gene-TIME-integrated clinical decision framework for immunotherapy stratification in non–small cell lung cancer.

The growing recognition that immunotherapy efficacy in NSCLC is influenced by the coordinated interaction between oncogenic drivers and the TIME has important implications for clinical decision-making. Rather than a uniform treatment paradigm, treatment selection may be optimized by tailoring immunotherapy to specific molecular and immune contexts across disease stages and treatment settings (74). **Figure 4** summarizes a pragmatic, gene–TIME–integrated framework that links molecular diagnostics and immune profiling to treatment strategies in locally advanced, metastatic, consolidation, perioperative, and refractory scenarios.

### 5.1 Systemic and consolidation immunotherapy in locally advanced and metastatic NSCLC: a gene–TIME–informed perspective

In advanced and metastatic NSCLC, ICIs have become a cornerstone of systemic therapy, either as monotherapy or in combination with chemotherapy and/or antiangiogenic agents. In parallel, consolidation immunotherapy following definitive chemoradiotherapy has reshaped the management of unresectable stage III disease, most notably with durvalumab based on landmark clinical trials.

From a targetable driver gene–TIME perspective, patients without actionable driver alterations and exhibiting immune-inflamed (“immune-hot”) TIME phenotypes represent the prototypical beneficiaries of ICI-based therapy. In these patients, first-line ICI monotherapy or ICI-based combinations are commonly selected and are supported by robust clinical activity and durable survival benefit in multiple studies (75). As illustrated in **Figure 4**, this subgroup occupies a treatment pathway in which immunotherapy forms the therapeutic backbone across advanced and consolidation settings.

In contrast, tumors harboring EGFR, ALK, or other actionable kinase-driven alterations often exhibit immune-cold or immune-excluded TIME states and derive limited benefit from ICIs. Moreover, certain treatment sequences involving ICIs and TKIs have been associated with a higher incidence of specific toxicities (e.g., pneumonitis or hepatotoxicity) in some reports, warranting cautious integration of immunotherapy in this population. Accordingly, targeted therapy remains the preferred systemic approach in advanced disease, while routine use of ICIs—either as monotherapy or consolidation—remains controversial and is generally restricted to carefully selected patients or clinical trials (76). **Figure 4** highlights this cautious positioning of immunotherapy in driver-positive disease, particularly in consolidation settings.

KRAS-mutant NSCLC occupies an intermediate and heterogeneous space, in which co-mutation context can meaningfully shape immune phenotype and ICI responsiveness. KRAS single-mutant tumors, especially those with inflamed TIME features, may respond favorably to ICIs or ICI-based combinations. Conversely, KRAS tumors co-mutated with STK11 or KEAP1 frequently display immunosuppressive or immune-excluded TIME states and are associated with reduced ICI responsiveness, suggesting that combination of chemotherapy, antiangiogenic therapy, or other immune-modulating strategies may be required, as reflected in the branching pathways shown in **Figure 4**.

In unresectable stage III NSCLC, whether gene–TIME information can refine patient selection for consolidation immunotherapy remains an open but clinically relevant question. While durvalumab is broadly applied, emerging data suggest that patients with unfavorable gene–TIME profiles may require closer surveillance or novel consolidation strategies, a concept incorporated into the stratification logic of **Figure 4**.

### 5.2 Perioperative immunotherapy in resectable NSCLC: insights from the targetable driver gene–TIME axis

The introduction of neoadjuvant and adjuvant immunotherapy, alone or combined with chemotherapy, has significantly altered the treatment landscape of resectable NSCLC, improving pathological response rates and reducing recurrence risk in selected patients (77). However, the applicability of perioperative immunotherapy across molecular subtypes remains an area of active debate.

From a gene–TIME perspective, patients with EGFR or ALK alterations represent a population in which perioperative immunotherapy remains controversial. Current guidelines and expert consensus diverge regarding the routine use of ICIs in these patients, given the limited immunogenicity of kinase-driven tumors and the availability of highly effective targeted therapies (78). In contrast, driver-negative tumors with immune-inflamed TIME characteristics appear particularly well suited for perioperative ICI-based approaches. In these patients, neoadjuvant or adjuvant immunotherapy—especially when combined with chemotherapy—may enhance pathological response and reduce recurrence risk, aligning with the favorable immune context depicted in **Figure 4**.

Importantly, the integration of TIME-related biomarkers into perioperative stratification may further refine patient selection. Beyond PD-L1 expression, immune infiltration patterns assessed by multiplex immunohistochemistry or digital pathology, as well as dynamic monitoring of circulating tumor DNA (ctDNA) before and after surgery, offer promising tools to identify patients at high risk of recurrence who may benefit from intensified immunotherapy or closer follow-up (79). Nevertheless, assay standardization, clinically actionable thresholds, and prospective validation across platforms and populations remain essential before broad routine adoption.

### 5.3 Treatment sequencing and rechallenge in refractory or relapsed disease: implications of the gene–TIME axis

In refractory or relapsed NSCLC, treatment options become increasingly limited, and therapeutic decisions must balance efficacy, toxicity, and patient-specific factors. In this context, the targetable driver gene–TIME axis provides valuable insights into treatment sequencing and rechallenge strategies.

For EGFR- or ALK-driven tumors progressing on TKIs, the use of ICIs—either immediately or after a treatment interval—has shown limited efficacy, and certain sequences of TKIs and ICIs have been associated with increased toxicity (e.g., pneumonitis or hepatotoxicity) in some reports. Gene–TIME profiling may help identify rare subsets with more permissive immune landscapes, but in general, immunotherapy rechallenge should be approached cautiously and preferably within clinical trials (80). Patients with KRAS tumors harboring STK11 or KEAP1 co-mutations face a particularly challenging scenario following ICI failure. In these cases, strategies aimed at remodeling the TIME—such as combinations with antiangiogenic agents, metabolic interventions, or novel immunomodulators—may be required (81). For heavily pretreated patients with limited but controllable disease burden, integrating gene–TIME information may also inform the balance between local therapies (e.g., radiotherapy or ablation) and continuation of systemic treatment. Integrating molecular and immune context can help tailor individualized treatment sequences rather than defaulting to uniform salvage approaches.

### 5.4 A simplified gene–TIME–traditional biomarker–integrated decision framework

Building on the preceding sections, a simplified and clinically applicable stratification framework integrating targetable driver genes, TIME characteristics, and traditional biomarkers can be proposed, as summarized in **Figure 4**.

The first step involves comprehensive molecular profiling using next-generation sequencing to identify actionable driver alterations and key co-mutations, such as STK11, KEAP1, or SMARCA4, thereby establishing the molecular foundation of the gene–TIME subtype. The second step incorporates assessment of TIME-related features, including PD-L1 expression, tumor mutational burden, immune infiltration patterns, and, where feasible, immune infiltration patterns and spatial immune architecture derived from digital pathology, multiplex immunohistochemistry, or multiplex imaging (82).

The final step integrates disease stage and clinical context—ranging from resectable versus unresectable disease to systemic treatment, consolidation, perioperative, or refractory settings—to assign patients to representative gene–TIME–context categories (83). These categories may help inform treatment selection, such as ICI monotherapy or combinations in immune-inflamed, driver-negative tumors; TKI-dominant approaches in driver-positive disease; or combination and trial-based strategies in immunotherapy-resistant KRAS co-mutant subtypes.

As illustrated in **Figure 4**, this framework does not aim to replace existing guidelines but rather to complement them by embedding molecular and immune biology into practical clinical pathways. By aligning oncogenic drivers, immune contexture, and clinical scenarios, the gene–TIME–based stratification model may offer a rational and flexible approach to precision immunotherapy in NSCLC. Prospective validation in biomarker-driven cohorts and trials will be essential before routine implementation.

## 6 Limitations

Despite the conceptual value of the driver gene–TIME axis in integrating oncogenic signaling with tumor immune contexture, several limitations should be acknowledged. First, a substantial proportion of the mechanistic insights summarized in this framework derive from preclinical models or transcriptomic analyses rather than prospective clinical studies. Second, considerable heterogeneity exists across published clinical cohorts with respect to patient populations, disease stage, treatment exposure, and analytical methodologies, which may contribute to variability in reported associations between driver gene subtypes and immune phenotypes. Third, commonly used immune biomarkers—including PD-L1 expression, tumor mutational burden, and immune infiltration patterns—are measured using different platforms, assays, and thresholds across studies, limiting cross-study comparability. Finally, co-mutation–defined subgroups (e.g., KRAS–STK11 or KRAS–KEAP1) are often underrepresented in individual cohorts, reducing statistical power and potentially affecting the stability of reported associations. Therefore, while the gene–TIME framework provides a useful conceptual model for interpreting molecular–immune interactions in NSCLC, further validation in large prospective and biomarker-driven clinical studies will be essential before its routine clinical implementation.

## 7 Conclusion

NSCLC is a biologically heterogeneous malignancy characterized by intricate crosstalk between oncogenic driver alterations and the tumor immune microenvironment. Beyond dictating sensitivity to targeted therapies, accumulating evidence suggests that targetable driver genes can influence or reshape the TIME through coordinated regulation of oncogenic signaling, cytokine and chemokine networks, metabolic reprogramming, and immune cell recruitment and function. These driver gene–associated immune landscapes may exert a profound influence on immunotherapy responsiveness and resistance, thereby contributing to the substantial variability in clinical outcomes observed across molecular subtypes. Viewing NSCLC through the integrative lens of the “targetable driver gene–TIME axis” unifies molecular pathology with immunobiology, provides a coherent framework for interpreting prognostic heterogeneity and differential treatment responses, and offers a biologically grounded rationale for refined immunotherapy stratification, while recognizing that this model remains conceptual and requires further prospective validation.

Future efforts should focus on translating this conceptual framework into clinically actionable strategies. Large-scale, multicenter cohorts and prospective clinical trials are needed to validate the association between distinct driver gene–TIME subtypes and immunotherapy outcomes across disease stages and treatment settings. The integration of emerging technologies—including single-cell and spatial transcriptomics, digital pathology, and longitudinal circulating tumor DNA monitoring—holds promise for capturing the spatial and temporal dynamics of immune contexture with greater precision. Such multidimensional profiling is particularly critical for high-risk subgroups, including EGFR/ALK-driven tumors and KRAS cancers harboring immune-resistant co-mutations, in which conventional immunotherapy approaches have shown limited efficacy. Rationally designed combination regimens and optimized treatment sequencing guided by the driver gene–TIME axis should be systematically evaluated in biomarker-driven studies. Ultimately, embedding molecular and immune stratification into routine clinical decision-making will be essential for transforming the gene–TIME axis from a conceptual paradigm into a practical foundation for precision immunotherapy in NSCLC.
